# Variants of human papillomaviruses 16 (HPV16) in Uigur women in Xinjiang, China

**DOI:** 10.1186/s13027-016-0089-2

**Published:** 2016-08-18

**Authors:** Hongchang He, Hongtao Li, Peiwen Fan, Junling Zhu, Zhenzhen Pan, Huan Pan, Dan Wu, Xianxian Ren, Xiaoqing Guo, Dongmei Li, Zemin Pan, Renfu Shao

**Affiliations:** 1 Department of Biochemistry and Molecular Biology, School of Medicine, Shihezi University, Xinjiang Endemic and Ethnic Disease and Education Ministry Key Laboratory, Shihezi, Xinjiang 832002 China; 2 Department of Pathology, the First People Hospital of Kashgar, Kashgar, Xinjiang 844000 China; 3 Genecology Research Centre, Faculty of Science, Health, Education and Engineering, University of the Sunshine Coast, Maroochydore DC, Queensland 4558 Australia

**Keywords:** HPV16, Uigur women, Cervical cancer

## Abstract

**Background:**

Persistent infection of high-risk human papillomaviruses 16 (HPV16) has been considered as the leading cause of cervical cancer. In this study we assessed HPV16 sequence variation and genetic diversity of HPV16 variants in cervical cancer in Uigur women in Xinjiang, China. We analyzed the nucleotide sequences of the open reading frames of E6 and E7, and part of the open reading frames of L1 of HPV16 in Uigur women.

**Methods:**

Biopsies of histologically confirmed HPV16 infections with cervical cancer were obtained from 43 Uigur women in Xinjiang, China. E6, E7 and L1 genes of HPV16 of all samples were amplified and sequenced; the sequences were used in phylogenetic analysis of HPV16 variants.

**Results:**

Our analysis revealed nine nucleotide changes in E6 (five changes), E7 (one change) and L1 (three changes) gene. The most frequently observed variations were T350G (79.1 %). One variation T295G (D64E) at E6 were detected in 6 cases (KT959536, KT959542, KT959546, KT959550, KT959553, KT959558). Deletion (464Asp) along with insertion (448Ser) were observed in L1 (100 %). Most variants were European lineage (97.7 %); only one belongs to Asia variants with common T178G (D25E) in E6 and A647G (N29S) in E7.

**Conclusion:**

The most prevalent HPV16 variants in the Uigur women we studied were of the European lineage. Our results indicate that HPV16 European lineage may serve as a harmful factor associated with the development and progression of cervical cancer.

**Electronic supplementary material:**

The online version of this article (doi:10.1186/s13027-016-0089-2) contains supplementary material, which is available to authorized users.

## Background

Cervical cancer was the third most common cancer among women in the world, with 527,624 new cases and 265,653 deaths in 2012 [1]. Cervical cancer is relatively common in China. Uigur women in Xinjiang, China, have one of the highest incidence of cervical cancer (527/100000) in the world [2] and are often diagnosed in young women [3].

Persistent infection by high-risk HPV16, has been recognized as a critical etiological factor for cervical cancer [4], which is present in over half of invasive cervical cancer cases worldwide [5, 6]. In Xinjiang, China, HPV16 was the most prevalent type [7].

Multiple factors are involved in the development and progression of cervical cancer and most HPV16 infection can be removed by the immune system but a small proportion can progress to cervical cancer. Previous studies demonstrated that HPV16 variants increased the risk for progression to cervical cancer [8–10], but the roles of HPV16 genetic variation are poorly understood.

Based on the genomic analysis of HPV16, seven major lineages of HPV16 variants have been detected and are related to geographic areas: European (E), Asian (As), Asian American (AA), African 1 (Af1), African 2 (Af2), North American (NA) [11] and a recently discovered Javanese variant (Java) in Indonesia [12]. The relative risk of each HPV16 variant for cervical cancer may be population dependent; each variant also differs in potential oncogenicity and geographical distribution [13–16]. However, much less is known about the epidemiology of HPV16 variants and their association with cervical cancer in Uigur women in Xinjiang, China.

Several mutations in E6 and E7, L1 genes may have great influence on the efficiency of infection, viral antigenicity and immunogenicity. A number of studies have suggested that the non-European variants have an increased risk for progression to high-grade squamous intraepithelial lesions (HSIL) when compared with European variants [9, 10, 17]. The E6 and E7 viral oncogenes are consistently present in all stages of HPV-mediated cervical cancers and interact with cellular proteins tightly linked to several signaling pathways [18]. Moreover, Almajhdi et al. demonstrated that oncoproteins E6 and E7 could be considered as promising targets for prophylactic HPV vaccine [4]. The major L1 capsid proteins have the property to self-assemble into virus-like particles (VLPs), which generated protective effects by immunization against papillomavirus disease [19, 20] and can be used as an ideal target for immunotherapeutic approaches against HPV-induced cervical cancer [21–23].

In Xinjiang, previous research showed that HPV16 was the most prevalent HPV type in Uigur women [24]. We analyzed the nucleotide sequences of E6, E7 and L1 genes from cervical cancer to investigate the diversity of HPV16 variants and evaluate the risks of HPV16 variants associated with cervical cancer in Uigur women in Xinjiang, China.

## Methods

### Sample collection

Biopsies of histologically confirmed HPV16 infections with cervical cancer were obtained from 43 Uigur women, who attended the People Hospital of Kashi (southern Xinjiang) and the People Hospital of Autonomous region (northern Xinjiang) during the years 2011 to 2014. 20 of the 43 women were residents of the southern Xinjiang, and the other 23 were in the northern Xinjiang. All of the cases were identified as squamous carcinomas. The diagnosis of histopathological grades was examined independently by two gynecologic pathologists. Tissues were stored at 4 °C no more than 24 h after surgical removal and subsequently cut into small fragments and stored in liquid nitrogen for genomic DNA extraction. Informed consent was obtained from all patients and the study protocol was reviewed and approved by the ethics committees of the hospitals.

### DNA extraction and typing of HPV

DNA was extracted from the 43 cervical samples with the SK1252 Genomic DNA Isolation kit (Shanghai Sangon Biological Engineering Technology and Services Company) according to the manufacturer’s instruction. HPV16 DNA was identified by polymerase chain reaction (PCR) using HPV16-specific primers (Table 1).

**Table 1:** Primers for HPV16 detection, amplification and sequencing

	Primer name	Primer sequence	Gene area covered (bp)
HPV16 detection	pHPV16 E6-F	5′-GACCCAGAAAGTTACCACAG-3′	146-374
	pHPV16 E6-R	5′-CACAACGGTTTGTTGTATTG-3′	
E6/E7	16E6-15 N	5′-AAACTAAGGGCGTAACCGAAATC-3′	44-910
	16E7-16C	5′-CAGCCTCTACATAAAACCATCCAT-3′	
	16E6-13 N	5′-AACCGAAATCGGTTGAACCG-3′	60-857
	16E7-13C	5′-TGCAGGATCAGCCATGGTAGAT-3′	
	MY11-N	5′-GCMCAGGGWCATAAYAATGG-3′	6602-7013
L1	MY09-C	5′-CGTCCMARRGGAWACTGATC-3′	
	GP-N	5′-CTGTGGTWGATACYACWCGCAGTAC-3′	6656-7013

*F* forward primer, *R* reverse primer, *N* normal strand, *C* complementary strand. Degenerate primers used : M = A/C; R = A/G; W = A/T; Y = C/T

### PCR amplification and sequencing

Fragments of HPV16 E6, E7 and L1 were amplified by PCR (Table 1). Each PCR was 50 μl containing 20 pmoles of each primer, 50 mM KCl, 2.5 mM MgCl_2_, 100 mM Tris–HCl, pH 8.3, 0.1 % Triton X-100, 50 μM of each dNTP, 1.8 U of HotStar Taq polymerase (QIAGEN) and 5 μl template DNA. The cycling conditions were: 94 °C for 5 min; 30 cycles of 55 °C for 45 s, 72 °C for 60 s, 94 °C for 15 s, 55 °C for 45 s, 72 °C for 5 min. Sequencing primers were listed in Table 1.

### Phylogenetic analysis of HPV16 variants

PCR products were purified using SAP (Promega) and Exo I (Epicentre) and sequenced directly using ABI BigDye Terminator v3.1 Cycle Sequencing Kit on a DNA analyzer (ABI3130XL) at the Genesky Biotechnologies Inc (Shanghai, China). Single Nucleotide Polymorphisms (SNP) were analyzed by software Polyphred and were aligned with the prototype (GenBank: NC_001526.2) [25] and other variants, As (GenBank: AF534061; AB889492), Af-1 (GenBank: AF472508; HQ644238), AA (GenBank: AF402678), and AA1 (GenBank: HQ644247).

**Fig. 1:**
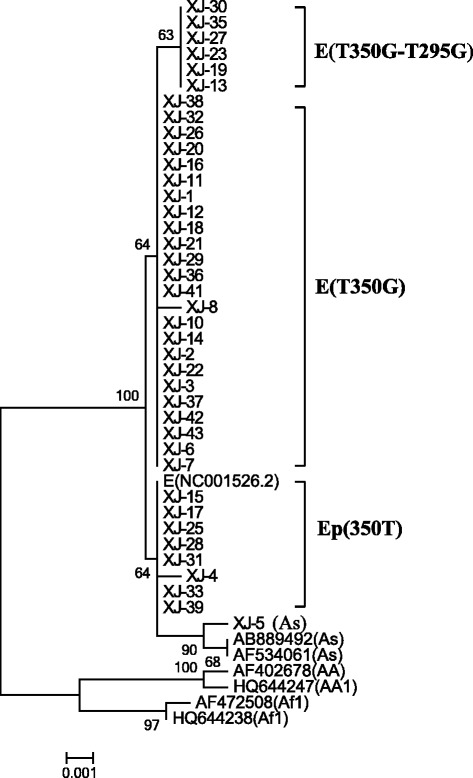
Phylogenetic studies were performed on a combined E6-E7-partial L1 nucleotide sequence alignment of 1137 positions from each case, which was constructed by the neighbor joining method and the Kimura 2-Parameter model by MEGA 6 package. Bootstrap proportions were calculated with 1000 replicates. Study sequences are labeled in XJ numbers, others are reference GenBank sequences. E, European variant; Ep, European prototype; As, Asia lineage; AA, Asian American lineage; Af, African lineage

The sequences of E6, E7 and L1 genes were subsequently assembled together using Sequence Matrixv 1.7.8 (http://dx.doi.org/10.1111/j.1096-0031.2010.00329.x). Phylogenetic trees were built by MEGA 6 [26] (Fig. 1).

### GenBank accession numbers

The sequences generated in this study were deposited in GenBank with accession numbers KT959524 to KT959566 for E6, KT966608 to KT966650 for E7 and KT966651 to KT966689 for L1 genes.

## Results

Forty-three full-length E6, E7 and 39 partial L1 genes (nt 6659–7023) were successfully amplified and sequenced. Sequences obtained were compared to the HPV16 prototype reference (European prototype, NC_001526.2). Gene sequence variation in E6, E7 and partial L1 genes of HPV16 were shown in Table 2.

**Table 2:** Nucleotide sequence variations in HPV16 of E6, E7, L1 ORFs, lineage classification and predicted amino acid changes

					E6			E7		L1	
HPV16Varians			G96A	A131C	T178G	T295G	T350G	A647G	6901-6902	6951-6953	A6989G
Amino acid changes					D25E	D64E	L83V	N29S	448insSer	464delAsp	
E Referene			G	A	T	T	T	A	insATC/GTC	delGAT	A
AA			G	A	T	T	G	A	ATC	GAT	A
As			G	A	G	T	T	G	ATC	GAT	A
Af			G	A	T	T	T	A	ATC	GAT	A
		Prevalence									
XJ-4	EP	No.(%)							ATC	GAT	g
XJ-25	EP	8 (18.6)							ATC	GAT	
XJ-28	EP								ATC	GAT	
XJ-15	EP								ATC	GAT	
XJ-17	EP								ATC	GAT	
XJ-31	EP								ATC	GAT	
XJ-33	EP								ATC	GAT	
XJ-39	EP								ATC	GAT	
XJ-1	E-350G	34 (79.1)					G		ATC	GAT	
XJ-2	E-350G						G		ATC	GAT	
XJ-3	E-350G						G		ATC	GAT	
XJ-6	E-350G						G		ATC	GAT	
XJ-7	E-350G						G		ATC	GAT	
XJ-8	E-350G		a				G		ATC	GAT	
XJ-9	E-350G						G		*	*	*
XJ-10	E-350G						G		ATC	GAT	
XJ-11	E-350G						G		GTC	GAT	
XJ-12	E-350G						G		ATC	GAT	
XJ-14	E-350G						G		ATC	GAT	
XJ-16	E-350G						G		ATC	GAT	
XJ-18	E-350G						G		ATC	GAT	
XJ-20	E-350G						G		ATC	GAT	
XJ-21	E-350G						G		ATC	GAT	
XJ-22	E-350G						G		ATC	GAT	
XJ-24	E-350G						G		ATC	GAT	
XJ-29	E-350G						G		ATC	GAT	
XJ-31	E-350G						G		ATC	GAT	
XJ-32	E-350G						G		ATC	GAT	
XJ-34	E-350G						G		*	*	*
XJ-36	E-350G						G		ATC	GAT	
XJ-37	E-350G						G		ATC	GAT	
XJ-38	E-350G						G		ATC	GAT	
XJ-40	E-350G						G		*	*	*
XJ-41	E-350G						G		ATC	GAT	
XJ-42	E-350G						G		ATC	GAT	
XJ-43	E-350G						G		ATC	GAT	
XJ-13	E-350G					G	G		ATC	GAT	
XJ-19	E-350G					G	G		ATC	GAT	
XJ-23	E-350G					G	G		ATC	GAT	
XJ-27	E-350G					G	G		ATC	GAT	
XJ-30	E-350G					G	G		ATC	GAT	
XJ-35	E-350G					G	G		ATC	GAT	
XJ-5	AS	1 (2.3)		c	G				ATC	GAT	
	Mutation	Prevalence (%)	2.3	2.3	2.3	14.0	79.1		100	100	2.4

Capital letters indicate variants with an amino acid change, Lower-case letters indicate silent mutations; The asterisk (*) indicates which segment failure to amplification

### E6 and E7 genes of HPV16

**Fig. 2:**
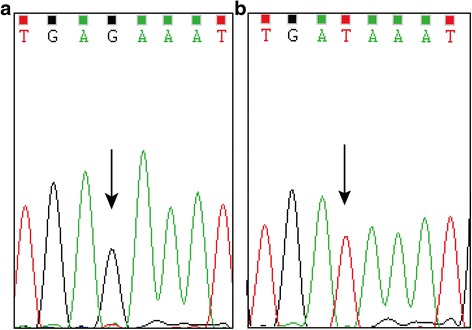
A sequencing electropherogram showing detected a novel nucleotide variation in E6 segment of HPV 16. (**a**) E6 T295G; (**b**) 295 T prototype. Variant spot indicated by arrow

Six nucleotide changes were observed in E6 and E7 (Table 2), which contained four missense mutations and two silent mutations. The four missense mutations, T178G, T295G, T350G in E6 and A647G in E7, result in amino acid changes aspartic acid to glutamic acid (D25E), aspartic acid to glutamic acid (D64E), leucine to valine (L83V), and asparagine to serine (N29S), respectively. The point mutations at nt 131 (A to C) and nt 96 (G to A) were silent mutations. The most frequently observed variations were T350G (34/43, 79.1 %) and T295G (6/43, 14.0 %). Point mutation T295G was a novel variation, which has not been reported before (Fig. 2). Co-variations of T350G and T295G were found in six cases (Table 2). In contrast to the high variation rate in E6, E7 gene was highly conserved in all samples, except for A131C, T178G and A647G, which were present in one sample.

### L1 gene of HPV16

Our sequence analysis showed that three base pair (ATC/GTC) were inserted into nt 6901–6902 along with three base pair (GAT) deleted at nt 6951–6953 in all samples (100 %); thus, a serine was inserted in amino acid (aa) position 449 whereas an asparagine was deleted from aa position 464. The sequence insertions and deletions of HPV16 L1 gene have not been reported before. The variant with the nucleotide insertion (GTC) at nt 6901–6902 was only observed in one sample. In addition, a silent change at nt 6989 (A to G) was observed in one sample (Table 2).

### Phylogenetic analysis

Sequence analysis of a combined E6-E7-partial L1 nucleotide sequence alignment revealed that all of the HPV16 variants identified were in the European lineage (97.7 %) (see Fig. 1; Additional file 1: Figures S1 and S2 for E6, E7 respectively in the supplemental material) except for one that was in the Asian lineage (AS), of which 8 out of 43 (19 %) to European prototype (350 T) and 34 out of 43 (81 %) were European variant (T350G). Of the 34 European variants, 28 (83.4 %) were the European variant (T350G). All of the 39 L1 sequences are in the European (E) lineage (see Additional file 1: Figure S3 in the supplemental material); none is in the Asian-American (AA) or African (Af) lineage.

## Discussion

Epidemiological data suggest that variants of the same HPV type are biologically distinct and may confer differential pathogenic risks [8]. Hence, understanding the distribution of HPV16 variants is of great significance for designing regional vaccines. Several studies reported that the distribution of HPV16 variants among Chinese women were highly similar. Several lines of evidence indicated that most HPV16 variants were of Asian and European lineage [14, 27–29]. However, the distribution of HPV16 variants among Uigur women was much less studied.

In this study, we showed that the most prevalent HPV16 variant type in Xinjiang was the European lineage. In contrast, Asian lineage, which is prevalent in other regions in China, was absent in the Uigur women. It may be because of ethnically specific of Uigur. No variants in AA, NA, Af and Javanese lineages were observed. These results raised the possibility that European lineage has a preferential role in progression to malignancy and is associated with the development of invasive cervical cancer in Uigur women in Xinjiang.

Based on our results, the European lineage consisted of cases of 350 T prototype, cases of variant 350G (L83V), and 1 case of Asia variant. HPV16 E6 L83V variant is prevalent in high-grade lesions and is associated with progression of cervical malignancy in Moroccan [30]; this variant was more prevalent than HPV16 E6 prototype 350 T in women with persistent infection and cervical disease progression [31, 32]. The functional implication of the L83V substitution requires more studies. In addition, novel nucleotide variation (T295G) is found in E6 gene of European variant in 6 cases, along with 350G mutation. Our results suggested that co-variations of T350G -T295G may be a specific characteristic of a newly potential sublineage within HPV16 European lineage in Uigur women of Xinjiang.

In good agreement with previous reports [22, 33, 34], the current study showed that E7 region was strongly conserved as compared to E6. We found only one mutation A647G (N29S), which is common in Asia women. One survey showed that amino acid change N29S in the E7 was frequent in cervical cancers [28] and is associated with a higher monogenic risk in Korean women [35]. Mutations at the Cys-X-X-Cys motifs showed that this region contributed to the transforming potential of E7 [36]. Similar mutants described by Alan et al. [37] showed a decreased ability to transform BRK cells.

Surprisingly, at the L1 gene, three nucleotide variations were found. One silent and two novel nucleotide variations are found; three base pair (ATC/GTC) were inserted at nt 6901–6902 along with 3 base pair (GAT) deleted at nt 6951–6953 in all cases (Table 2). This lead to a serine inserted in amino acid position 449 and an asparagine deleted from amino acid position 464. Any change may affect the efficiency of infection and viral antigenicity of the L1 protein. Additionally, this feature of L1 may be used to distinguish the European (E) and Non-European (NE) variants of HPV16. The structure and characteristics of the nucleotide variations and their functional implications require further investigations.

## Conclusion

We investigated the genetic variation of HPV16 in Uigur women in Xinjiang. Our results show that HPV16 variant of European lineage is the most common type in Uigur women in Xinjiang, which is markedly different from elsewhere in China. Future studies should expand into larger population of patients to evaluate the association between HPV16 variants and the risk for cervical cancer, and to understand the evolution of HPV16 variants in Uigur women in Xinjiang.

## Abbreviations

AA, Asian American; Af1, African 1; Af2, African 2; As, Asian; D, aspartic acid; DNA, deoxyribonucleic acid; E, European; E, glutamic acid; HPV, human papillomaviruses; HPV16, human papillomaviruses 16; HSIL, high-grade squamous intraepithelial lesions; Java, Javanese variant; L, leucine; N, asparagine; NA, North American; NE, Non-European; PCR, polymerase chain reaction; S, serine; V, valine; VLPs, virus-like particles

## Additional file

Additional file 1: Phylogenetic trees of HPV16 E6, E7 and L1 variants based on Neighbor Joining Molecular Phylogenetic analysis. **Figure S1.** Neighbor Joining Molecular Phylogenetic analysis using 43 nucleotide sequences of HPV16 E6 gene. Phylogenetic studies were performed on E6 nucleotide sequence alignment of 477 positions from each case, which was constructed by the neighbor joining method and the Kimura 2-Parameter model by MEGA 6 package. Bootstrap proportions were calculated with 1000 replicates. Study sequences are labeled in KT GenBank accession numbers, others are reference GenBank sequences. E, European variant; Ep, European prototype; As, Asia lineage; AA, Asian American lineage; Af, African lineage. **Figure S2.** Neighbor Joining Molecular Phylogenetic analysis using 43 nucleotide sequences of HPV16 E7 gene. Phylogenetic studies were performed on E7 nucleotide sequence alignment of 297 positions from each case, which was constructed by the neighbor joining method and the Kimura 2-Parameter model by MEGA 6 package. Bootstrap proportions were calculated with 1000 replicates. Study sequences are labeled in KT GenBank accession numbers, others are reference GenBank sequences. E, European variant; Ep, European prototype; As, Asia lineage; AA, Asian American lineage; Af, African lineage. **Figure S3.**  Neighbor Joining Molecular Phylogenetic analysis using 39 nucleotide sequences of HPV16 L1 gene. Phylogenetic studies were performed on partial L1 nucleotide sequence alignment of 369 positions from each case, which was constructed by the neighbor joining method and the Kimura 2-Parameter model by MEGA 6 package. Bootstrap proportions were calculated with 1000 replicates. Study sequences are labeled in KT GenBank accession numbers, others are reference GenBank sequences. E, European variant; Ep, European prototype; As, Asia lineage; AA, Asian American lineage; Af, African lineage. (PDF 136 kb)
