# Supplementary material for: Variants of human papillomaviruses 16 (HPV16) in Uigur women in Xinjiang, China
Source: Infect Agent Cancer. 2016 Aug 18;11:44. doi: 10.1186/s13027-016-0089-2 (PMC4989296; doi:10.1186/s13027-016-0089-2)
Supplement: Additional file 1: — Phylogenetic trees of HPV16 E6, E7 and L1 variants based on Neighbor Joining Molecular Phylogenetic analysis. Figure S1. Neighbor Joining Molecular Phylogenetic analysis using 43 nucleotide sequences of HPV16 E6 gene. Phylogenetic studies were performed on E6 nucleotide sequence alignment of 477 positions from each case, which was constructed by the neighbor joining method and the Kimura 2-Parameter model by MEGA 6 package. Bootstrap proportions were calculated with 1000 replicates. Study sequences are labeled in KT GenBank accession numbers, others are reference GenBank sequences. E, European variant; Ep, European prototype; As, Asia lineage; AA, Asian American lineage; Af, African lineage. Figure S2. Neighbor Joining Molecular Phylogenetic analysis using 43 nucleotide sequences of HPV16 E7 gene. Phylogenetic studies were performed on E7 nucleotide sequence alignment of 297 positions from each case, which was constructed by the neighbor joining method and the Kimura 2-Parameter model by MEGA 6 package. Bootstrap proportions were calculated with 1000 replicates. Study sequences are labeled in KT GenBank accession numbers, others are reference GenBank sequences. E, European variant; Ep, European prototype; As, Asia lineage; AA, Asian American lineage; Af, African lineage. Figure S3. Neighbor Joining Molecular Phylogenetic analysis using 39 nucleotide sequences of HPV16 L1 gene. Phylogenetic studies were performed on partial L1 nucleotide sequence alignment of 369 positions from each case, which was constructed by the neighbor joining method and the Kimura 2-Parameter model by MEGA 6 package. Bootstrap proportions were calculated with 1000 replicates. Study sequences are labeled in KT GenBank accession numbers, others are reference GenBank sequences. E, European variant; Ep, European prototype; As, Asia lineage; AA, Asian American lineage; Af, African lineage. (PDF 136 kb) [file 13027_2016_89_MOESM1_ESM.pdf]

Supplemental material

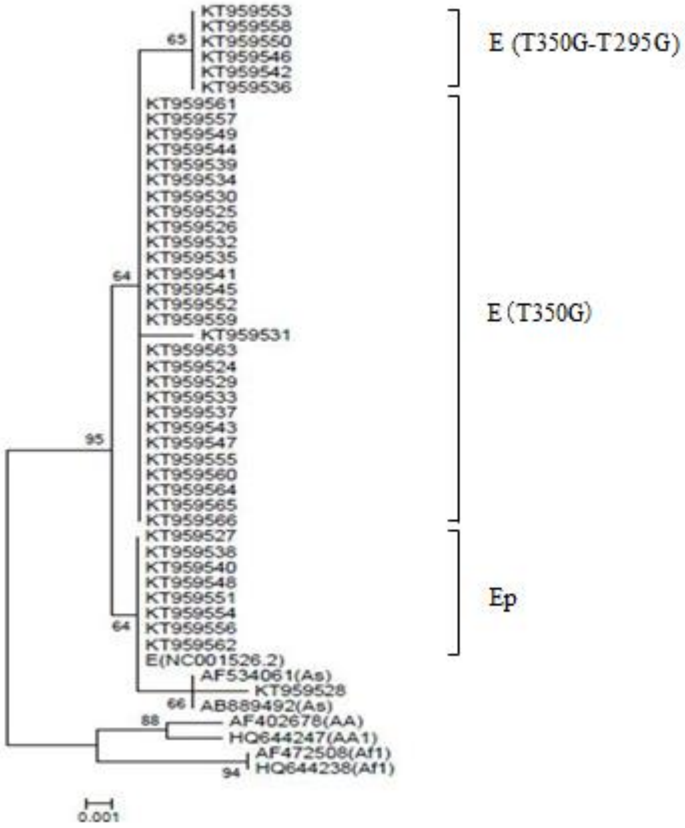

Figure S1 Phylogenetic studies were performed on E6 nucleotide sequence alignment of 477 positions from each case, which was constructed by the neighbor joining method and the Kimura 2-Parameter model by MEGA 6 package. Bootstrap proportions were calculated with 1000 replicates. Study sequences are labeled in KT GenBank accession numbers, others are reference GenBank sequences. E, European variant; Ep, European prototype; As, Asia lineage; AA, Asian American lineage; Af, African lineage.

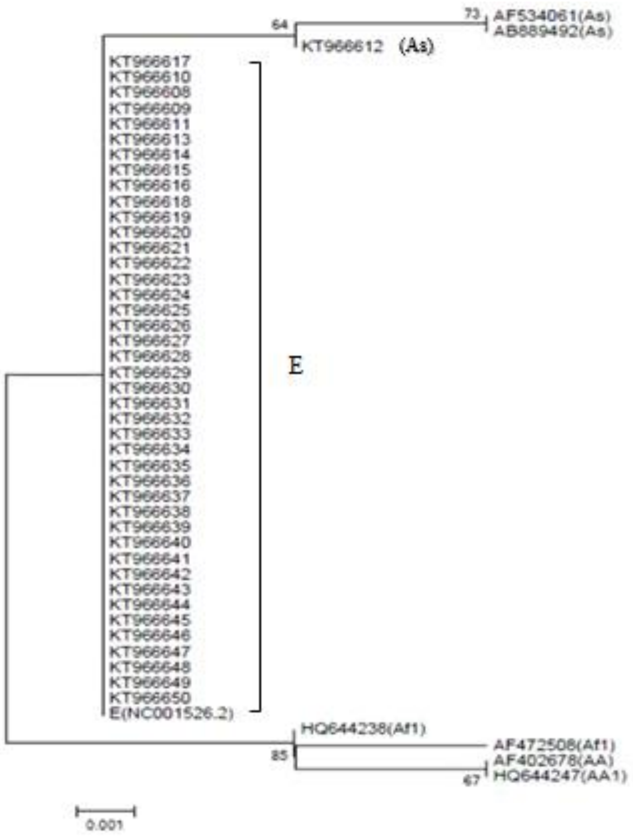

Figure S2 Phylogenetic studies were performed on E7 nucleotide sequence alignment of 297 positions from each case, which was constructed by the neighbor joining method and the Kimura 2-Parameter model by MEGA 6 package. Bootstrap proportions were calculated with 1000 replicates. Study sequences are labeled in KT GenBank accession numbers, others are reference GenBank sequences. E, European variant; Ep, European prototype; As, Asia lineage; AA, Asian American lineage; Af, African lineage.

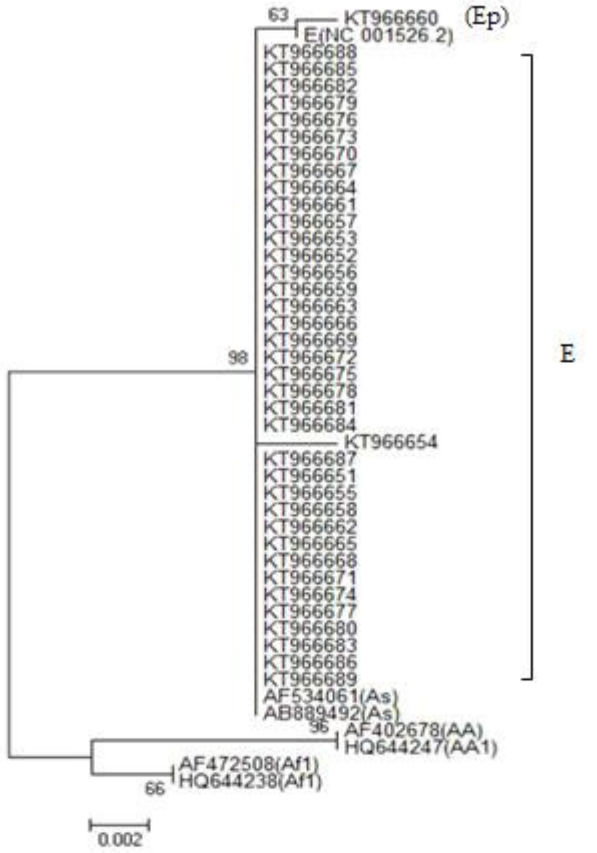

Figure S3 Phylogenetic studies were performed on partial L1 nucleotide sequence alignment of 369 positions from each case, which was constructed by the neighbor joining method and the Kimura 2-Parameter model by MEGA 6 package. Bootstrap proportions were calculated with 1000 replicates. Study sequences are labeled in KT GenBank accession numbers, others are reference GenBank sequences. E, European variant; Ep, European prototype; As, Asia lineage; AA, Asian American lineage; Af, African lineage.
